# The Rab-Rabphilin system in injured human podocytes stressed by glucose overload and angiotensin II

**DOI:** 10.1152/ajprenal.00077.2020

**Published:** 2020-06-22

**Authors:** Olga Martinez-Arroyo, Ana Ortega, Javier Perez-Hernandez, Felipe J. Chaves, Josep Redon, Raquel Cortes

**Affiliations:** ^1^Cardiometabolic and Renal Risk Research Group, INCLIVA Biomedical Research Institute, Valencia, Spain; ^2^Genomics and Diabetes Unit, INCLIVA Biomedical Research Institute, Valencia, Spain; ^3^CIBER of Diabetes and Associated Metabolic Diseases, Institute of Health Carlos III, Minister of Health, Barcelona, Spain; ^4^Internal Medicine Unit, Hospital Clínico Universitario, Valencia, Spain; ^5^CIBER of Physiopathology of Obesity and Nutrition, Institute of Health Carlos III, Minister of Health, Madrid, Spain

**Keywords:** angiotensin II, glucose, kidney injury, podocyte, Rab-Rabphilin system

## Abstract

Kidney injury in hypertension and diabetes entails, among in other structures, damage in a key cell of the glomerular filtration barrier, the podocyte. Podocytes are polarized and highly differentiated cells in which vesicular transport, partly driven by Rab GTPases, is a relevant process. The aim of the present study was to analyze Rab GTPases of the Rab-Rabphilin system in human immortalized podocytes and the impact of high glucose and angiotensin II. Furthermore, alterations of the system in urine cell pellets from patients with hypertension and diabetes were studied. Apoptosis was analyzed in podocytes, and mRNA level quantification, Western blot analysis, and immunofluorescence were developed to quantify podocyte-specific molecules and Rab-Rabphilin components (Rab3A, Rab27A, and Rabphilin3A). Quantitative RT-PCR was performed on urinary cell pellet from patients. The results showed that differentiated cells had reduced protein levels of the Rab-rabphillin system compared with undifferentiated cells. After glucose overload and angiotensin II treatment, apoptosis was increased and podocyte-specific proteins were reduced. Rab3A and Rab27A protein levels were increased under glucose overload, and Rabphilin3A decreased. Furthermore, this system exhibited higher levels under stress conditions in a manner of angiotensin II dose and time treatment. Immunofluorescence imaging indicated different expression patterns of podocyte markers and Rab27A under treatments. Finally, Rab3A and Rab27A were increased in patient urine pellets and showed a direct relationship with albuminuria. Collectively, these results suggest that the Rab-Rabphilin system could be involved in the alterations observed in injured podocytes and that a mechanism may be activated to reduce damage through the vesicular transport enhancement directed by this system.

## INTRODUCTION

Kidney injury, characterized by a progressive decrease in glomerular filtration rate and/or an increase in urinary albumin excretion (UAE) (10, 40), is a frequent complication associated with diabetes mellitus (DM) and hypertension (HTN) (58, 67). Despite efforts to prevent and treat it, the incidence of renal injury is increasing, often associated with alterations in the glomerular filtration barrier, in which podocytes play a central role in preserving structure and function (53, 63). Podocytes are well-differentiated and highly polarized cells with an elaborate cytoarchitecture (15). Due to the complexity of their structure and function, the mechanisms involved in their injury are still not fully understood, but their involvement in the pathophysiology of renal diseases has been previously described (41, 46, 68). In this regard, in vitro studies have revealed that exposure to glucose and angiotensin II (ANG II) induces alteration of specific proteins and activation of intracellular pathways leading to podocyte injury (35, 56).

One of the most important processes in podocytes is membrane trafficking, essential for maintaining cell polarization and for close cell-to-cell communication. Altered membrane trafficking may be the cause and/or result of podocyte injury. In this sense, Rab proteins, a group of monomeric small GTPases, are key counterparts in regulating vesicle formation, controlling both the specificity and directionality of intracellular transport (12, 47, 71). They are critical regulators of secretory vesicle exocytosis and have been widely described in synaptic neurons (13, 33). Specifically, recent literature reports that the Rab proteins Rab3A and Rab27A cooperate to achieve vesicle exocytosis by recruiting specific effector proteins such as Rabphilin3A to drive vesicle docking at target membranes (48).

However, despite its important function in vesicular traffic, studies analyzing the presence of the Rab-Rabphilin system (Rab3A, Rab27A, and their effector Rabphilin3A) in the kidney are scarce (2, 14, 49, 50). Previous studies by our group have identified an association between the *RPH3A* gene polymorphism, codifying for Rabphilin3A, and risk of UAE (39). A previous study (23) showed the association of the same polymorphism with UAE in the Framingham cohort. However, further research is needed on the as-yet unclear implications of this system in podocyte injury in the context of DM and HTN.

In the present study, we sought to investigate the presence of the Rab-Rabphilin system in cultured human podocytes and analyze changes in their expression when challenged with elevated glucose concentrations and ANG II. Additionally, we aimed to detect and quantify Rab GTPases mRNA levels in urinary samples from patients with HTN and DM and their association with UAE levels.

## MATERIALS AND METHODS

### Immortalized Human Podocyte Cell Culture

Conditionally human immortalized podocytes (cell line: AB8/13) were kindly provided by Prof. Moin Saleem (Children’s Renal Unit and Academic Renal Unit, University of Bristol, Southmead Hospital, Bristol, UK). Particularly in this cell line, at the “permissive” temperature of 33°C, the SV40 T antigen is active, maintaining cells in an undifferentiated proliferative state. Thermoswitching them to the “nonpermissive” 37°C temperature silences the transgene, and the cells become growth arrested and differentiated (51). Briefly, podocytes were cultured on collagen-coated flasks or plates in RPMI-1640 with 10% FBS (Biowest), 1% penicillin-streptomycin, and 1% insulin‐transferrin‐selenium (GIBCO, ThermoFisher Scientific). Cells were propagated at 33°C until 80% confluence was reached and were trypsinized and replated at proper density. Next, they were allowed to differentiate at 37°C with a lower FBS concentration (2%) for 10–14 days. All treatment experiments were performed on differentiated podocytes starting at *day 10* of differentiation and with a confluence of 80%. Cells were serum starved and then treated with normal glucose (NG; 5.5 mM d-glucose) and high glucose (HG; 30 mM d-glucose) concentrations for 72 h. Differentiated podocytes were also treated with increasing concentrations (0.1, 1, and 2 µM) of ANG II (Sigma-Aldrich) for 6, 24, and 48 h for Western blot analyses, 24 h for quantitative RT-PCR, and 48 h for flow cytometry and immunofluorescence analyses.

### Homogenization of Cell Pellets, Gel Electrophoresis, and Western Blot Analysis

Total protein was extracted from podocyte cell pellets using RIPA buffer (ThermoFisher Scientific) with added protease inhibitor supplements (Sigma-Aldrich). After centrifugation, supernatants were collected and protein content was determined by the Lowry method using BSA as the standard. Homogenates were loaded into NuPAGE 4–12% polyacrylamide gels or NuPAGE 3–8% Tris-acetate gels (Invitrogen) and were then transferred to PVDF membranes for Western blot analysis. After being blocked overnight with 1% BSA, membranes were incubated with primary antibodies at room temperature for 2 h. Membranes were washed three times with Tris‐buffered saline (TBS) with Tween 20 (TBS-T; 20 mM Tris·HCl, 150 mM NaCl, and 0.1% Tween 20) and incubated with alkaline phosphatase‐conjugated anti‐rabbit IgG, anti‐mouse IgG, or anti‐guinea pig IgG antibodies (Sigma-Aldrich) at room temperature for 1 h. After membranes were washed three times with TBS-T and TBS, the chromogen 5‐bromo‐4‐chloro‐3‐indolyl phosphate/nitroblue tetrazolium (Sigma-Aldrich) was used to detect bound antibodies. Finally, bands were visualized using an imaging system and quantified by the TotalLab TL-100 (version 2008) program. The primary antibodies used were rabbit polyclonal anti-synaptopodin (1/1,000, Sigma-Aldrich), guinea pig polyclonal anti-nephrin (1/400, OriGene), rabbit polyclonal anti-Wilms’ tumor-1 (WT-1; 1/500, Abcam), rabbit polyclonal anti-CD2-associated protein (CD2AP; 1/500, Invitrogen), mouse monoclonal anti-Rab3A (1/100, Sigma-Aldrich), mouse monoclonal anti-Rab27A (1/500, Abcam), and rabbit polyclonal anti-Rabphilin3A (1/500, Sigma-Aldrich). Monoclonal anti-β-actin antibody (1/6,000, Sigma-Aldrich) was used as a loading control.

### Optical and Confocal Microscopy Imaging

A Leica DMi1 optical microscope (Wetzlar) and Leica LAS (version 4.9) camera software were used to follow the podocyte differentiation process. Images were taken at different stages of differentiation and also during podocyte treatments. Moreover, measures and morphological parameters were analyzed in treated podocytes (membrane blebbing and area and circularity of cells) using ImageJ software (version 1.46 r, National Institutes of Health). For this analysis, measurements were taken from 150 cells of each experimental group in random and nonoverlapping fields of three independent replicates.

For immunofluorescence analysis, samples were fixed in 4% paraformaldehyde for 15 min at room temperature, washed in cold PBS, and blocked in PBS + BSA (1%) for 30 min at room temperature. After being blocked, samples were incubated overnight at 4°C with the same primary antibodies as for the Western blot analyses: anti-synaptopodin (1/40), anti-nephrin (1/50), anti-Rab3A (1/80), anti-Rab27A (1/50), and anti-Rabphilin3A (1/50) in PBS-Tween 20 (PBS-T) + BSA (1%) buffer. Next, they were incubated in darkness with Alexa-conjugated secondary antibody (Invitrogen) for 1 h at room temperature in the same buffer. Within the last half an hour of the secondary antibody incubation, PBS-T + BSA (1%) with DAPI (1/1,000) was added to identify nuclei. After incubation, samples were washed for 3 × 5 min with 1% PBS + BSA. Cell apoptosis of treated podocytes was assessed using DAPI and determining chromatin condensation, nuclei fragmentation, and nuclear condensation of podocytes following the protocol described by Cummings et al. (5) and analyzing at least 250 cells/sample. Analysis of the F-actin cytoskeleton was assessed by phalloidin staining on differentiated podocytes treated with HG and ANG II concentrations. Cells were fixed in 4% paraformaldehyde for 15 min at room temperature, washed three times with PBS, permeabilized with 0.1% Triton X-100, washed, and incubated with phalloidin-iFluor 594 Reagent (1/1,000, Abcam) for 1 h at room temperature. Within the last half an hour of the incubation period, cells were stained with DAPI. After incubation, cells were washed three times in PBS. Finally, fluorescence samples were observed with Leica confocal microscope DMi8 (Wetzlar), and images were processed using ImageJ (version 1.46 r, National Institutes of Health) software. F-actin fiber orientation was measured with the FibriTool plugin (ImageJ), which quantifies the orientation and anisotropy of fibrillary structures in raw images. Values close to 0 correlate to isotropic or no ordered fibers, and values close to 1 represent perfectly orientated filaments.

### RNA Isolation and cDNA Synthesis

RNA was extracted from podocyte and human urine pellets using the miRNeasy mini kit (Qiagen), based on spin column isolation and following the manufacturer’s instructions as previously reported (43). After extraction, RNA concentration and purity were confirmed by the 260-to-280-nm absorbance ratio using a NanoDrop 2000 spectrophotometer (ThermoFisher Scientific). Total RNA was reverse transcribed to cDNA using Ready-To-Go You-Prime First-Strand Beads (GE Healthcare) following the protocol indications. cDNA samples were stored at −80°C until use.

### Quantitative RT-PCR

Quantitative RT-PCR was performed using Qiagen Multiplex PCR Master Mix with LC Green reagent (Qiagen) in the LightCycler 480 II real-time PCR system (Roche). A seminested PCR was performed specifically for the Rabphilin3A gene (*RPH3A*) to improve the sensitivity and specificity of cDNA amplification. Briefly, this process involved the use of two primer sets and two successive PCRs. The first set of primers was designed to anneal to sequences upstream from the second set of primers and was used in the initial PCR. Amplicons resulting from the first PCR were used as a template for a second set of primers (using the same 3′ primer as the 3′ primer used in the first PCR) and a second amplification step. For *RPH3A* amplicon identification, we performed high-resolution capillary electrophoresis using QIAxcel (Qiagen), and fragment length was assessed through QIAxcel software. A neuronal cell line (SH-SY5Y, CRL2266, American Tissue Type Collection) was used as a positive control of *RPH3A* expression. The Primer3 program was used to design the corresponding primers of target genes, which are shown in Table 1. All assays were run in triplicate, including appropriate controls and the non-template control. Besides these, melting curve analysis was performed to evaluate the specificity of the amplicons generated. β-Actin (*ACTB*) and β_2_-microglobulin (*B2MG*) housekeeping genes were used to normalize podocyte and urine pellet gene expression. Finally, the relative amount for each target gene was calculated by the $2^{-\Delta \Delta {\text{C}}_{\text{t}}}$ comparative method (where C_t_ is threshold cycle).

**Table 1.** Quantitative RT-PCR primer sequences

Target (Gene Name)	Sequence	Size, bp
*SYNPO*	Forward: 5′-GCCGCAAATCCATGTTTACT-3′	20
	Reverse: 5′-CTCATCCGCTGTCTGTACCA-3′	20
*WT-1*	Forward: 5′-TTCGCAATCAGGGTTACAGC-3′	20
	Reverse: 5′-AATGAGTGGTTGGGGAACTG-3′	20
*NPHS1*	Forward: 5′-TGCAGTTTCCCCCAACTAAC-3′	20
	Reverse: 5′-ACGCTGACGCATGTCAAGT-3′	19
*CD2AP*	Forward: 5′-AGGCATGGGAATGTAGCAAG-3′	20
	Reverse: 5′-TGACGCTTCTTGGTCTTCTTC-3′	21
*RAB3A*	Forward: 5′-CCTCATGTATGACATCACCAACG-3′	23
	Reverse: 5′-CCTCAAAGAACTCGAACCCAAG-3′	22
*RAB27A*	Forward: 5′-AAAGAGTGGTGTACAGAGCCAGTG-3′	24
	Reverse: 5′-GTCGTTAAGCTACGAAACCTCTCC-3′	24
*RPH3A*	Forward: 5′-GTCAAGCTCTGGCTGAAACC-3′	20
	Reverse: 5′-GCAGCCTCCGATGTAATCAT-3′	20
*ACTB*	Forward: 5′-TGGAGAAAATCTGGCACCAC-3′	20
	Reverse: 5′-CATGATCTGGGTCATCTTCTCG-3′	22
*B2MG*	Forward: 5′-TCCAGCGTACTCCAAAGATTC-3′	21
	Reverse: 5′-GTCAACTTCAATGTCGGATGG-3′	21

*SYNPO*, synaptopodin; *WT-1*, Wilms’ tumor-1; *NPHS1*, nephrin; *CD2AP*, CD2-associated protein; *RAB3A*, Ras-related protein Rab-3A; *RAB27A*, Ras-related protein Rab-27A; *RPH3A*, Rabphilin-3A; *ACTB*, β-actin; *B2MG*, β_2_-microglobulin.

### Flow Cytometry

After glucose and ANG II treatments, podocyte cell pellets were trypsinized, resuspended, centrifuged for 3 min at 1,300 rpm, and washed with sterile PBS. They were then resuspended in 200 µL annexin V binding buffer (Immunostep, Salamanca, Spain) and incubated in darkness for 15 min at room temperature with 5 µL annexin V and 5 min with 7 µL 7-amino-actinomycin D. Finally, 400 µL of annexin binding buffer were added. A total of 10,000 events from each replicate were analyzed by flow cytometer (BD LSRFortessa X-20) with FACSDiva 8.0.1 software (BD Bioscience, Franklin Lakes, NJ).

### Human Subjects and Urine Samples

The study population included 64 patients with essential HTN, 30 of them with DM, recruited from the Internal Medicine area of Hospital Clínico Universitario of Valencia, Spain. HTN was defined according to the European Society of Hypertension (systolic blood pressure > 140 mmHg and/or diastolic blood pressure > 90 mmHg) (67), and DM was defined according to the World Health Organization (1). Among all patients, 10 patients were diagnosed also with DM (DM no UAE) and 20 patients with DM and increased UAE (DM UAE). Twenty patients were nondiabetic patients (no DM no UAE), and 14 patients nondiabetic with increased UAE (no DM UAE). Table 2 shows clinical characteristics of the patient groups. All patients received antihypertensive treatment at the time of study, and patients with DM were treated with oral antidiabetic agents. The study protocol was approved by the Ethics Committee of the Hospital Clínico Universitario of Valencia in accordance with the Declaration of Helsinki of 1975 as revised in 2013 (69). All patients gave written informed consent.

**Table 2.** Clinical characteristics of patient groups

Variables	No DM		DM	
	Increased UAE	No UAE	Increased UAE	No UAE
*n*	14	20	20	10
Age, yr	50.79 ± 9.27	54.45 ± 6.04	60.30 ± 9.33	54.78 ± 4.15
Sex (men/women), %	64.3/35.7	60.0/40.0	75.0/25.0	77.8/23.2
Systolic blood pressure, mmHg	130.79 ± 10.59	134.05 ± 18.55	143.05 ± 23.61	140.78 ± 34.06
Diastolic blood pressure, mmHg	82.50 ± 8.86	88.00 ± 13.53	82.95 ± 17.10	89.00 ± 18.04
Glucose, mg/dL	97.43 ± 17.46	103.65 ± 9.51	154.150 ± 58.49^h^	149.11 ± 59.53^i^
Glycated hemoglobin, %	5.75 ± 0.07	5.62 ± 0.21	7.15 ± 1.17^g^	6.47 ± 1.05^i^
Total cholesterol, mg/dL	204.86 ± 35.98^d^	184.00 ± 22.66	184.60 ± 31.14^b^	150.67 ± 25.18^i^
LDL, mg/dL	133.43 ± 30.13	116.85 ± 19.74	114.05 ± 27.92^a^	86.00 ± 20.58^i^
HDL, mg/dL	56.00 ± 13.80	51.30 ± 10.85	42.20 ± 10.68	43.67 ± 8.60
Triglycerides, mg/dL	113.86 ± 44.12	121.40 ± 57.47	244.2 ± 194.3^g^	159.0 ± 65.02
Plasma creatinine, mg/dL	0.91 ± 0.34	0.85 ± 0.17	1.00 ± 0.41	0.99 ± 0.27
Glomerular filtratration rate, mL·min^−1^·1.73 m^−2^	92.04 ± 29.28	89.58 ± 16.97	86.74 ± 30.23	82.81 ± 23.51
Body mass index, kg/m^2^	29.46 ± 5.00	30.60 ± 6.49	35.10 ± 7.57	29.51 ± 4.66
Obesity, %	42.9	40.0	65.0	44.4
Dyslipidemia, %	85.7	75.0	95.0	100
UAE-to-creatinine ratio, mg/g	221.7 ± 229.7^f^	4.84 ± 6.45	307.3 ± 228.8^c^^,^ ^e^	3.03 ± 1.48

Values are means ± SD. DM, diabetes mellitus; UAE, urinary albumin excretion. Comparisons between diabetic groups:

a *P* < 0.05,

b *P* < 0.01, and

c *P* < 0.001. Comparisons between diabetic and nondiabetic groups:

d *P* < 0.05,

e *P* < 0.01, and

f *P* < 0.001. Comparisons between increased UAE groups:

g *P* < 0.05 and

h *P* < 0.01. Comparisons between no UAE groups:

i *P* < 0.05.

Patient fresh urine samples were taken from the first morning urine and were processed following our previously reported protocol (44). UAE was measured using a nephelometric immunoassay (Behring Institute). The level of albuminuria for each patient was considered as the mean value obtained from the morning spot urine samples and expressed as the albumin (mg)-to-creatinine (g) ratio (ACR). Increased UAE was defined as an ACR of ≥30 mg/g.

### Statistical Analysis

Results are expressed as fold changes and means ± SE for *n* independent observations, as indicated in the figures. A Kolmogorov-Smirnov test was used to analyze the normal distribution of the variables. Comparisons between two groups were performed with Student's *t* test or Mann-Whitney *U* test depending on data distribution, and Pearson's correlation coefficient was calculated to analyze the association between variables. The level of significance was set at *P* < 0.05. Statistical analyses were performed with SPSS software (version 20, SPSS), and graphical plots were created with SigmaPlot (Systat Software).

## RESULTS

### Identification of Differentiated Human Immortalized Podocytes and Podocyte-Associated Molecules

**Fig. 1.**
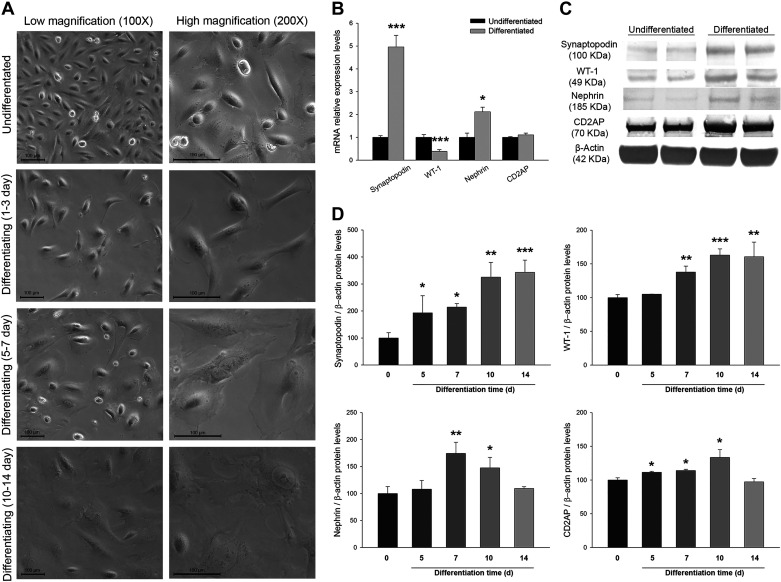
Identification of differentiated human immortalized podocytes. *A*: optical images at low (×100) and high (×200) magnifications of undifferentiated podocytes and at different stages of differentiation. *B*: relative mRNA expression levels of podocyte markers at differentiation stages compared with undifferentiated cells. *C*: Western blots of podocyte markers showing undifferentiated and differentiated podocyte cell pellets. WT-1, Wilms’ tumor-1; CD2AP, CD2-associated protein. *D*: bar graphs representing protein levels of podocyte markers comparing undifferentiated and differentiated podocytes at different days of differentiation. Data are expressed as means ± SE; *n* = 8 each group. mRNA levels were normalized to two housekeeping genes, and relative expression was expressed as fold changes, calculated by the following equation: $2^{-\Delta \Delta {\text{C}}_{\text{t}}}$ (where Ct is threshold cycle); undifferentiated group values were set to one-fold. Protein levels were previously normalized to β-actin and expressed as arbitrary units; undifferentiated group values were set to 100. Differentiation time is expressed in days (d). Scale bar = 100 µm. *P < 0.05, **P < 0.01, and ***P < 0.001 vs. the undifferentiated group.

At the morphological level, proliferating podocytes showed small cell bodies and an epithelial-like cobblestone shape when cultured at 33°C (undifferentiated state). After thermoswitching to 37°C for 10–14 days, they were differentiated into arborized cells, characterized by enlarged cell bodies with foot processes and spindle-like projections and junctions (Fig. 1*A*).

Additionally, we analyzed mRNA and protein levels of differentiation and podocyte-specific markers. We observed that synaptopodin and nephrin mRNA levels were higher in differentiated podocytes (4.96-fold increase, *P* < 0.001, and 2.12-fold increase, *P* < 0.05, respectively), WT-1 levels were lower (2.58-fold decrease, *P* < 0.001), and CD2AP showed no significant changes compared with the undifferentiated state (Fig. 1*B*).

Differentiated podocytes showed elevated protein levels compared with proliferating podocytes in synaptopodin (2.49-fold increase, *P* < 0.01), WT-1 (1.47-fold increase, *P* < 0.001), nephrin (1.49-fold increase, *P* < 0.05), and CD2AP (1.20-fold increase, *P* < 0.05) podocyte-associated molecules (Fig. 1*C*). When we compared the levels of these markers along differentiation times, we observed a general increase in all of them (Fig. 1*D*).

### Expression of the Rab-Rabphilin System in Undifferentiated and Differentiated Podocytes

**Fig. 2.**
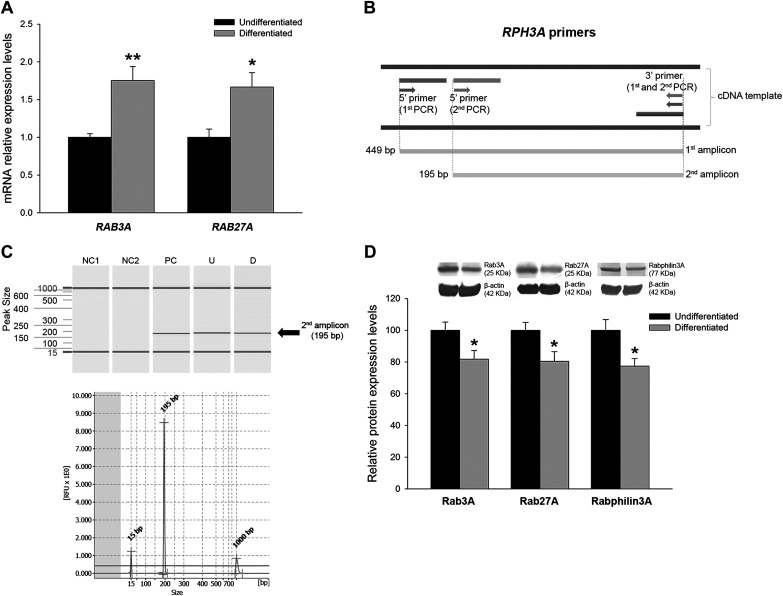
Analysis of Rab GTPases in differentiated podocytes. *A*: relative mRNA expression levels of *RAB3A* and *RAB27A* genes at differentiation stages compared with undifferentiated cells. *B*: Rabphilin3A gene (*RPH3A*) primer sets for seminested PCR experiments. *C*: capillary electrophoresis software capture of fragments belonging to the second PCR amplicon of *RPH3A*. The images show clear bands and peaks without any other fragment contamination. *D*: protein levels of Rab3A, Rab27A, and Rabphilin3A in differentiated podocytes compared with undifferentiated podocytes. Data are expressed as means ± SE; *n* = 8 each group. mRNA levels were normalized to two housekeeping genes, and relative expression was expressed as fold changes, calculated by the following equation: $2^{-\Delta \Delta {\text{C}}_{\text{t}}}$ (where C_t_ is threshold cycle); undifferentiated group values were set to one-fold. Protein levels were previously normalized to β-actin and expressed as arbitrary units; undifferentiated group values were set to 100. NC1, negative control of the first PCR; NC2, negative control of the second PCR; PC, positive control neuronal cell line; U, undifferentiated podocyte; D, differentiated podocyte; RFU, relative fluorescence units. *P < 0.05 and **P < 0.01 vs. the undifferentiated group.

The presence and differential expression of the Rab-Rabphilin subfamily was analyzed in human immortalized podocytes. *RAB3A* and *RAB27A* mRNA levels were higher in differentiated podocytes (1.75-fold increase, *P* < 0.01, and 1.66-fold increase, *P* < 0.05, respectively; Fig. 2*A*). Focusing on the *RPH3A* gene, we had to perform a seminested PCR technique with two primer sets to detect its expression in human podocytes, as previously reported (50) (Fig. 2*B*). We were able to detect *RPH3A* amplicons in both undifferentiated and differentiated podocytes, as shown by capillary electrophoresis captures performed to identify and measure fragment length (Fig. 2*C*).

Differentiated cells showed a reduction in protein levels of Rab3A (1.22-fold decrease, *P* < 0.05), Rab27A (1.25-fold decrease, *P* < 0.05), and Rabphilin3A (1.30-fold decrease, *P* < 0.01; Fig. 2*D*).

### Impact of HG and ANG II in Human Podocytes

Apoptosis-induced mRNA and protein levels of podocyte-specific molecules and the Rab-Rabphilin system were analyzed after glucose overload and addition of ANG II in cultured human podocytes.

#### Apoptosis and actin cytoskeleton rearrangements induced by podocyte treatments.

**Fig. 3.**
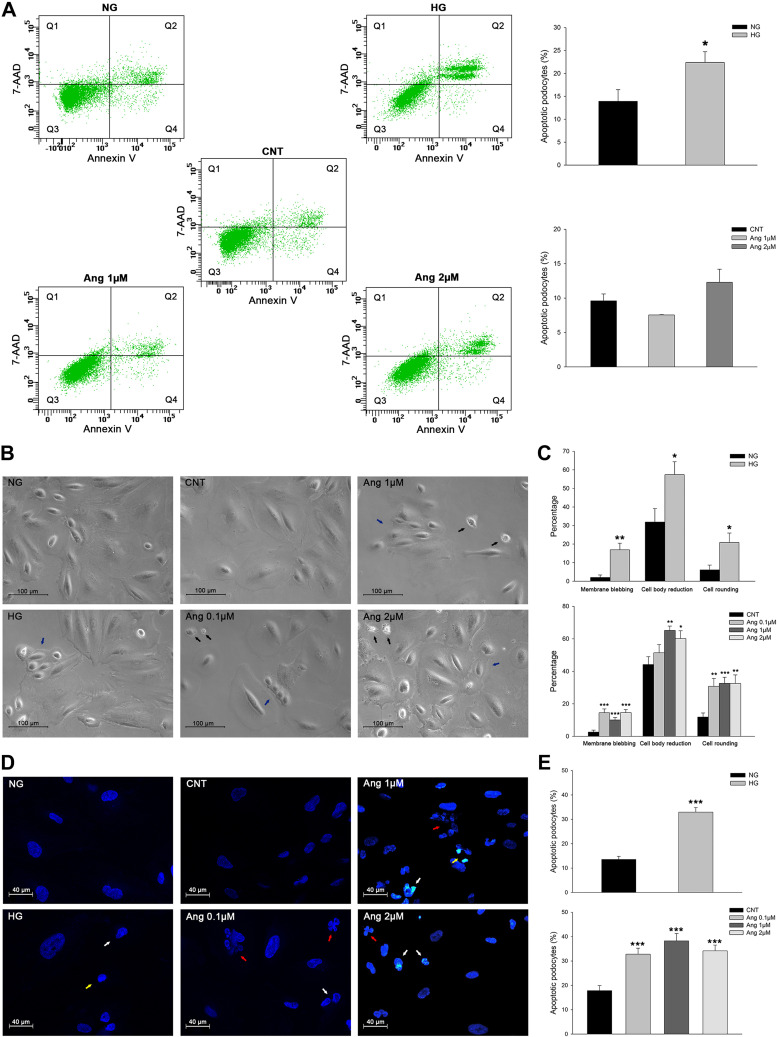
Podocyte apoptosis during glucose overload and ANG II treatment. *A*: flow cytometry images of annexin V/7-amino-actinomycin D (7-AAD) double staining-labeled cells (*n* = 3 per group) and graphs of the analysis performed. The number of apoptotic or necrotic cells was quantified by FACS analysis. The cytograms of early apoptotic cells, positive for annexin V but with still intact cell membranes (7-AAD negative), are shown in quadrant 4 (Q4). Cells with advanced stages of apoptosis or necrotic were both annexin V and 7-AAD positive and are shown in the quadrant 2 (Q2). Viable cells that did not bind annexin V or 7-AAD are depicted in quadrant 3 (Q3). Cells with lost intact cell membranes that bound 7-AAD and excluded annexin V are shown in quadrant 1 (Q1). NG, normal glucose; HG, high glucose; CNT, control. *B*: representative optical images showing morphological changes of podocytes subjected to glucose and ANG II treatments. Black arrows show membrane blebbing; blue arrows show cell body reduction and rounding. *C*: bar graphs representing the quantification of podocyte morphological parameters under both treatments. *D*: representative confocal images of DAPI staining showing nuclei alterations in apoptotic cells subjected to glucose and ANG II treatments; NG and CNT groups showed typical nuclei DAPI staining. White arrows show chromatin condensation, yellow arrows show nuclear condensation, and red arrows show nuclear fragmentation. *E*: bar graphs of apoptotic cells under glucose and ANG II treatments. Data are expressed as means ± SE. Scale bars = 100 µm in *B* and 40 µm in *D*. *P < 0.05, **P < 0.01, and ***P < 0.001 vs. the NG or CNT group.

Apoptosis was analyzed in human immortalized podocytes subjected to glucose overload and ANG II treatments by different approaches. Analyses by flow cytometry showed an increase of apoptosis in the HG group compared with the NG group (22.4% vs. 13.9%, *P* < 0.05) and a trend towards this under the highest ANG II treatment (2 µM) (12.3% vs 9.6%, *P* = 0.33; Fig. 3*A*).

Podocyte apoptosis studied by optical microscopy revealed that both treatments, HG and ANG II, induced typical apoptosis morphological alterations, such as membrane blebbing, cell volume reduction, and rounding (Fig. 3, *B* and *C*). Additionally, cell apoptosis analyses by DAPI staining of nuclei showed different parameters indicative of apoptosis increased in HG and ANG II-treated podocytes (Fig. 3, *D* and *E*).

F-actin fiber staining by rhodamine phalloidin staining was also performed in podocytes treated with elevated glucose and ANG II concentrations. In control podocytes, F-actin was distributed homogenously in bundles along the cell. Exposure to treatments induced cytoskeleton rearrangements that showed a disorganization of fibers evidenced by the anisotropy measurements performed, with reduced values in treated cells compared with control cells. We also observed more grouped F-actin fibers in peripheral regions of treated podocytes (see Supplemental Fig. S1 in the Supplemental Material, available online at https://doi.org/10.6084/m9.figshare.12443282).

#### Podocyte-specific molecule and Rab-Rabphilin system levels under glucose overload.

**Fig. 4.**
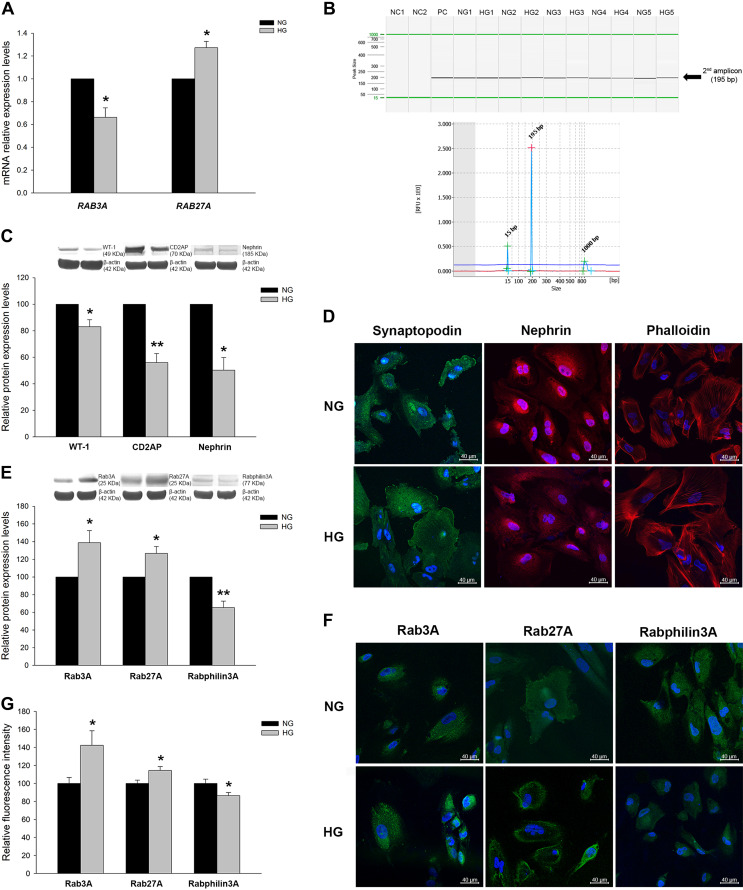
Podocyte markers and Rab GTPases expression under glucose treatment. *A*: mRNA levels of Rab GTPases under glucose treatments. *B*: capillary electrophoresis capture of Rabphilin3A gene (*RPH3A*) amplicon under glucose treatments. NC1, negative control of the first PCR; NC2, negative control of the second PCR; PC, positive control neuronal cell line. *C*: protein levels of podocyte markers under normal gluocose (NG) and high glucose (HG) conditions. WT-1, Wilms’ tumor-1; CD2AP, CD2-associated protein. *D*: confocal images of podocyte markers and F-actin fibers staining in NG- and HG-treated podocytes. *E*: protein levels of Rab GTPases under NG and HG treatments. *F*: confocal images of Rab GTPases in NG- and HG-treated podocytes. *G*: bar graph showing fluorescence quantification in NG and HG treatments of Rab GTPases. Data are expressed as means ± SE; *n* = 5 each group. mRNA levels were normalized to two housekeeping genes, and relative expression was expressed as fold changes, calculated by the following equation: $2^{-\Delta \Delta {\text{C}}_{\text{t}}}$ (where C_t_ is cycle threshold); NG group values were set to one-fold. Protein levels were previously normalized to β-actin and expressed as arbitrary units; NG group values were set to 100. Blue DAPI staining shows nuclei. Scale bars = 40 µm. *P < 0.05 and **P < 0.01 vs. the NG group.

When we analyzed mRNA levels under HG, the podocyte-associated molecules synaptopodin, WT-1, nephrin, and CD2AP did not reach statistically significant differences (data not shown); however, interestingly, the Rab-Rabphilin system was altered. *RAB3A* showed lower levels (1.51-fold decrease, *P* < 0.05) while *RAB27A* showed higher levels (1.27-fold increase, *P* < 0.05) in the HG group (Fig. 4*A*). A nested PCR of the *RPH3A* gene in podocytes subjected to glucose overload demonstrated its expression in treated podocytes as well. We detected the correct amplicon by capillary electrophoresis, but no quantification was performed (Fig. 4*B*).

Regarding protein changes, reduced levels of the podocyte markers WT-1 (1.20-fold decrease, *P* < 0.05), CD2AP (1.79-fold decrease, *P* < 0.01), and nephrin (1.99-fold decrease, *P* < 0.05) as well as a trend in synaptopodin (1.19-fold decrease, *P* > 0.05) were observed (Fig. 4*C*). These results were also confirmed by immunofluorescence in which under NG conditions, synaptopodin displayed a cytoplasmic pattern, extending its expression through podocyte foot processes, and nephrin showed a perinuclear expression pattern. Under HG conditions, synaptopodin revealed a rearranged pattern, not observed under NG conditions, and nephrin showed increased cytoplasmic expression and reduced at the perinuclear region; overall, its protein levels were reduced compared with NG conditions (Fig. 4*D*).

Interestingly, Rab GTPase proteins also showed protein changes under HG conditions. Rab3A and Rab27A revealed increased protein levels (1.39-fold increase and 1.27-fold increase, *P* < 0.05, respectively), whereas Rabphilin3A had reduced levels (1.54-fold decrease, *P* < 0.01; Fig. 4*E*). In immunofluorescence experiments, Rab3A, Rab27A, and Rabphilin3A displayed cytoplasm and membrane dotted expression patterns as expected in vesicular proteins, reaching also foot process extensions, and their levels were similar to those observed by Western blot analysis. Interestingly, under HG conditions, Rab27A revealed a change in its expression pattern compared with NG conditions, showing increased levels close to the podocyte plasma membrane (Fig. 4, *F* and *G*).

#### Podocyte-specific molecules and Rab-Rabphilin system levels under ANG II treatment.

**Fig. 5.**
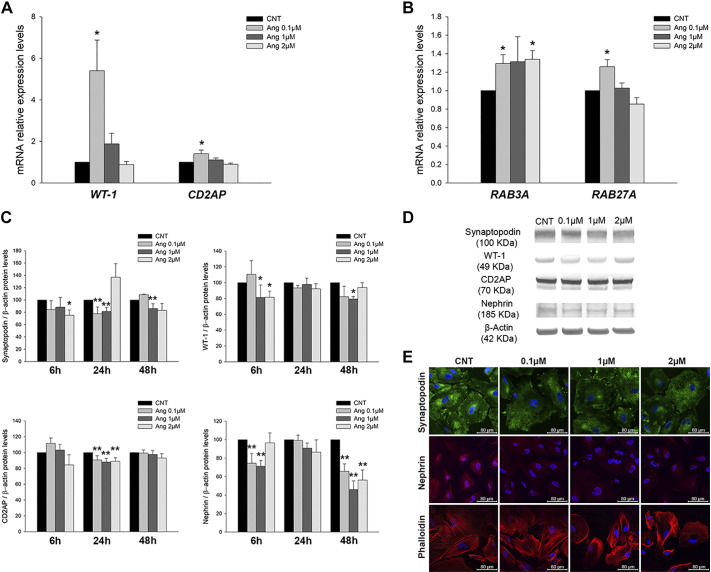

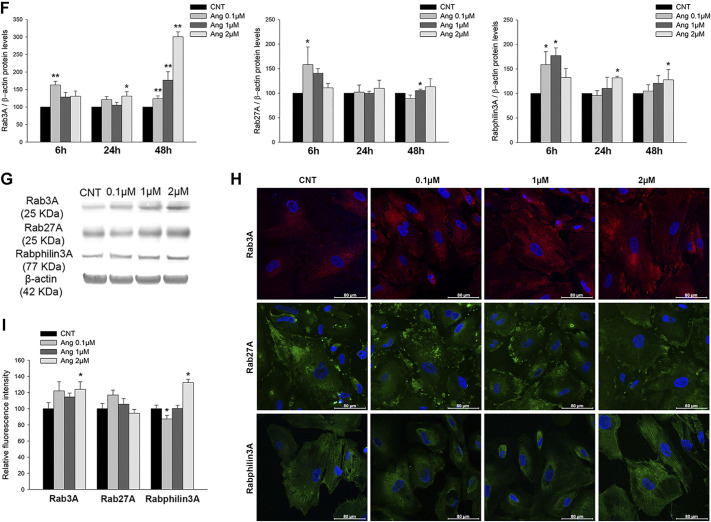
Podocyte markers and Rab GTPase expression under ANG II treatment. *A*: mRNA levels of podocyte markers under ANG II treatments. CNT, control; WT-1, Wilms’ tumor-1; CD2AP, CD2-associated protein. *B*: mRNA levels of Rab GTPases under ANG II increasing doses. *C*: protein levels of podocyte markers in CNT and ANG II treatments. *D*: representative membranes of podocyte markers under ANG II treatment. *E*: confocal images of podocyte markers synaptopodin and nephrin and F-actin fiber staining under ANG II treatments. *F*: protein levels of Rab GTPases in CNT and increasing concentrations of ANG II at 6, 24, and 48 h of treatment. *G*: representative Western blots of Rab GTPase levels under ANG II treatments. *H*: confocal images of Rab GTPases in ANG II-treated podocytes. *I*: bar graph showing the fluorescence quantification of Rab3A, Rab27A, and Rabphilin3A under ANG II treatments. Data are expressed as means ± SE; *n* = 5 each group. mRNA levels were normalized to two housekeeping genes, and relative expression was expressed as fold changes, calculated by the following equation: $2^{-\Delta \Delta {\text{C}}_{\text{t}}}$ (where C_t_ is cycle threshold); CNT group values were set to one-fold. Protein levels were previously normalized to β-actin and expressed as arbitrary units; CNT group values were set to 100. Blue DAPI staining shows nuclei. Scale bars = 80 µm. *P < 0.05 and **P < 0.01 vs. the CNT group.

With the addition of ANG II, mRNA quantification revealed higher levels of WT-1 (5.40-fold increase, *P* < 0.05) and CD2AP (1.41-fold increase, *P* < 0.05) only at the lowest ANG II concentration (Fig. 5*A*). Likewise, *RAB3A* and *RAB27A* mRNA levels were increased (Fig. 5*B*).

At the protein level, the podocyte-associated molecules synaptopodin, WT-1, nephrin, and CD2AP showed generally lower levels with ANG II-increased concentrations (Fig. 5, *C* and *D*). These observations were also reflected in immunofluorescence experiments, showing reduced synaptopodin expression and a rearranged pattern and lower levels of nephrin, with less perinuclear staining (Fig. 5*E*).

The Rab proteins (Rab3A, Rab27A, and Rabphilin3A) showed mainly increased expression during treatments and also showed differences according to ANG II doses and time treatments (Fig. 5, *F* and *G*). After ANG II treatment, cells were immunostained, and fluorescence analyses showed similar alterations in concordance with changes at the protein level, with increased Rab3A, Rab27A, and Rabphilin3A signals under ANG II stimulation. Interestingly, as was observed with glucose treatment, Rab27A showed increased membrane expression under ANG II treatment (Fig. 5, *H* and *I*).

### Levels of the Rab-Rabphilin System in Human Urine Samples From Patients With HTN and Relationship With UAE

**Fig. 6.**
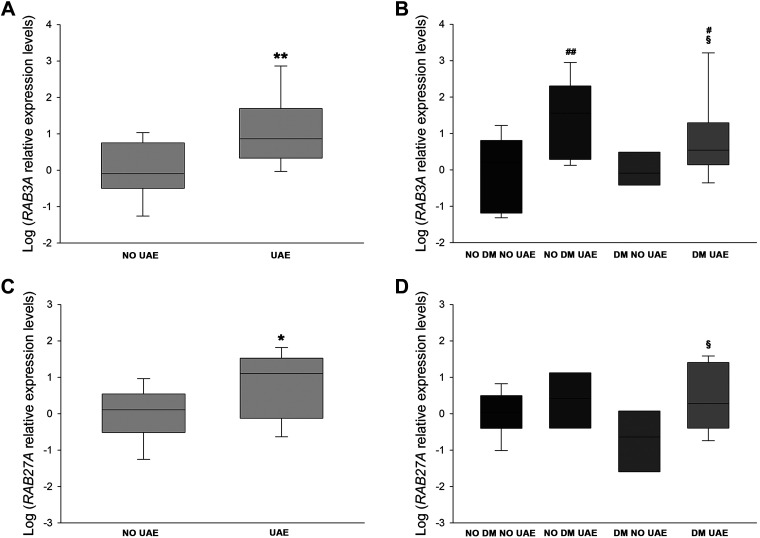
Urinary levels of Rab GTPases in patients with hypertension. *A* and *B*: box plots of mRNA levels of *RAB3A* in patients with hypertension with (*n* = 34) and without (*n* = 30) increased urinary albumin excretion (UAE; *A*) or divided into four groups (*B*). *C* and *D*: box plots of mRNA expression levels of *RAB27A* in patients with hypertension with (*n* = 34) and without (*n* = 30) increased UAE (*C*) or divided into four groups (*D*). Horizontal lines represent medians ± SE. mRNA levels were normalized to two housekeeping genes, , and relative expression was expressed as fold changes, calculated by the following equation: $2^{-\Delta \Delta {\text{C}}_{\text{t}}}$ (where C_t_ is cycle threshold). DM, diabetes mellitus. *P < 0.05 and **P < 0.01 vs. the no UAE group; #*P* < 0.05 and ##*P* < 0.01 vs. the no DM, no UAE group; §*P* < 0.05 vs. the DM, no UAE group.

Additionally, we analyzed the presence of mRNA levels of these Rab GTPases in urine pellets of patients with and without DM. Table 2 shows clinical characteristics of the patient groups. Higher *RAB3A* (2.17-fold increase, *P* < 0.01; Fig. 6*A*) and *RAB27A* levels (1.53-fold increase, *P* < 0.05; Fig. 6, *C* and *D*) were observed in the elevated UAE patient group than in the nonalbuminuric group. *RAB3A* was elevated in both patients without and with DM (2.56- and 2.34-fold increases, *P* < 0.01 and *P* < 0.05, respectively; Fig. 6*B*). Moreover, UAE levels were positively correlated with both *RAB3A* (*r* = 0.515, *P* < 0.05) and *RAB27A* (*r* = 0.475, *P* < 0.05).

## DISCUSSION

This study is the first to demonstrate the presence of Rab GTPases of the Rab-Rabphilin secretory system in human podocyte cell cultures and in human urinary samples. Aside from the changes observed in these molecules during podocyte differentiation, exposing them to cellular stresses such as HG and ANG II revealed notable alterations in Rab3A, Rab27A, and Rabphilin3A at gene and protein levels. Likewise, pellets from urine samples of patients with HTN and with and without incipient renal injury and/or DM showed increased expression of Rab mRNAs and association with incipient renal damage.

It is widely recognized that HTN and DM are primary causes of renal damage and that podocyte injury plays a major role in glomerular filtration barrier alterations, contributing to the progression of glomerular diseases (41, 68). Consequently, reducing podocyte damage has become a priority aim. The study of associated alterations subjecting podocyte cultures to stresses mimicking disease states is therefore a useful approach to elucidate new molecular players in kidney injury.

Consistent with previous reports (6, 31, 51), protein levels of podocyte-specific markers (synaptopodin, WT-1, nephrin, and CD2AP) increased in differentiated human podocytes, and changes under different treatments were also observed. Concerning mRNA levels, we detected an increase in all markers except for CD2AP and WT-1, showing reduced levels of WT-1 in differentiated podocytes. Other authors have described no WT-1 changes or slight increases during differentiation (6, 51), so our finding could be due to different compensatory regulation mechanisms for the increased protein levels at the end of the differentiation process. These discrepancies have been previously reported as being attributed to different half-lifes and production rates between mRNA and proteins and to a strong regulatory role for processes downstream transcription that can produce, among others, alterations in degradation rates of proteins (8, 34, 38, 66). Also important in these regulation mechanisms is the influence of small RNAs such as microRNAs, key regulators of gene expression (17).

Previous studies have highlighted the involvement of important podocyte metabolic pathways and alterations produced in cytoskeleton components in the onset and progression of renal diseases (22, 41, 45). In this study, we contribute to this research by showing reduced levels of podocyte-associated structural proteins under HG and ANG II treatments. Our group has previously demonstrated alterations in podocyte-specific markers in renal damage and urine from patients with HTN, revealing them as potential biomarkers of the disease (43, 44). Moreover, this study has brought novel insights to renal disease progression by deciphering new implications of the trafficking machinery encrypted by the Rab GTPase subfamily.

Most Rab GTPases studies are focused on the nervous system, and multiple research has associated these molecules with neurodegenerative disorders such as Alzheimer disease (3, 24, 27). In epithelia, Rab GTPases contribute toward generating polarity by regulating the trafficking of junctional proteins and by defining epithelial transport circuits and recycling endosomes (55). Rab GTPases are recognized by proteins called effectors in their GTP-bound state (29). Specifically, Rab3A plays a central role in neuron synaptic vesicle exocytosis (54, 61), a function carried out in cooperation with its effector Rabphilin3A, which interacts with cytoskeletal proteins (4, 7, 26). Additionally, Rabphilin3A also acts as an effector of Rab27A, whose main function has been described in the exosome secretion pathway (11, 28, 42). The secretory system enclosed by these three Rabs has previously been elucidated, establishing a relation between these proteins in the context of regulated secretion in exocytosis pathways (48, 65). In podocytes, vesicular trafficking directed by Rab GTPases represents a key process to maintain their polarized cell bodies, for foot processes formation, and also for the recycling and cell communication pathways (9, 59, 70). A study carried out by Giardino et al. (14) showed that Rab3A knockout produced exocytosis dysregulation and podocyte cytoskeleton alterations, supporting the importance of the Rab-Rabphilin system in vesicular transport of podocytes.

In differentiated podocytes, we showed a decrease of protein levels of Rab3A, Rab27A, and their effector Rabphilin3A and an increase in mRNA levels compared with the proliferative stage, suggesting a compensatory mechanism for the reduction observed, as explained above (8, 17, 34, 38, 66). Moreover, under HG and ANG II conditions, the decreased protein levels of podocyte differentiation markers together with the increased Rab3A and Rab27A protein levels (contrary to levels at differentiation) evidence that dedifferentiation may be occurring and that the Rab-Rabphilin system may be influencing on this process.

Podocyte dedifferentiation has been previously reported by our group in patients with HTN and lupus nephritis, in which we found viable podocytes secreted in urine and podocyte marker changes that suggested a dedifferentiation rather than apoptosis process (43, 44). Herman-Edelstein et al. (18) also observed this process in stressed human podocytes subjected to transforming growth factor-β and ANG II treatments, but, interestingly, our research points out novel players in this dedifferentiation: Rab3A, Rab27A, and Rabphilin3A membrane-trafficking GTPases.

The apoptosis assays performed on podocytes under HG and ANG II treatments also reinforced observations previously confirmed by other authors (16, 19, 20). Complementing these results, morphological analyses of treated podocytes showed an increase in apoptosis characteristic features in cells exposed to both treatments. We also revealed the presence of apoptotic nuclei in DAPI-stained micrographs, with nuclei and chromatin condensations. However, the lack of statistically significant changes in apoptosis assays under ANG II treatment surprisingly suggests that this might not be a question of marked apoptotic cell death but of initial dedifferentiation followed by cell death, as previously reported by other authors in human podocyte cultures (32, 52).

Additionally, F-actin fluorescence staining showed a cytoskeleton disorganization in podocytes subjected to HG and ANG II treatments and a grouping of fibers at the cell periphery and more diffuse in the cytoplasm. Alterations of podocyte structure have been widely observed in stress situations as glucose and ANG II overload and evidence the importance of these disruptions in podocyte injury (21, 25, 36, 37, 57, 64).

Immunofluorescence analyses revealed altered levels and distinct subcellular distributions of podocyte markers and Rab proteins under stressed conditions. Synaptopodin expression was rearranged under HG conditions, as previously observed (30). Nephrin expression changed from a perinuclear to more cytoplasmic pattern and was reduced under HG and ANG II exposure, similar to a previous study (62). These protein pattern changes and reduced expression also support that dedifferentiation occurred in these cells. Strikingly, the Rab GTPases images showed a particular Rab27A expression pattern. In contrast to the pattern observed in normal conditions with cytoplasmic distribution, a higher expression near the podocyte membrane was observed under HG and ANG II treatments. Taking into account Rab27A function in vesicle exocytosis, these observations suggest accentuated exosomal traffic under these stresses, an interesting area to explore in future research.

Consistent with our observations in podocyte cultures, we quantified *RAB3A* and *RAB27A* mRNAs in urine samples from patients with HTN and with and without DM and/or renal injury. Our findings showed an increase in both mRNA expression levels in patients with kidney disease and also a direct relationship between UAE levels and Rab mRNAs. In the case of *RAB3A*, this increase was significant in both albuminuric groups (with and without associated DM), which suggests that Rab3A could be involved or at least indicate the presence of incipient renal damage. This finding is consistent with the observation made by Rastaldi et al. (50), showing increased Rab3A in the glomerular tissue of a few patients with proteinuric diseases. Related to this, Rab3A knockout showed enhanced UAE excretion and glomerular and podocyte injury under HG (2, 14). Moreover, in Rab27A levels, this increase was statistically significant when analyzing both albuminuric groups together, suggesting that Rab27A plays a less potent but still important role in renal injury. Rab27A has also been studied at the renal level but has not been associated with albuminuria (60, 70).

In summary, in the present study, we demonstrate, for the first time, the presence of the Rab-Rabphilin system in both human urine samples and cultured human podocytes. The increase and different distribution patterns of the Rab-Rabphilin system in glucose- and ANG II treated podocytes indicates that enhanced vesicular trafficking through the Rab-Rabphilin system could be taking place in podocyte injury. In addition, we found important expression changes of this system in patients with HTN and DM with renal injury as well as an association between these Rab GTPases and albuminuria. Further experimental studies will be required to elucidate if modifying the Rab-Rabphilin system could result in risk reduction of kidney damage. Finally, our results suggest that Rab system GTPases may be orchestrating the alterations observed in podocytes during insults, conditioning the development and progression of renal damage.

## GRANTS

This work was supported by National Institutes of Health “Fondo de Investigaciones Sanitarias del Instituto de Salud Carlos III” {PI16/01402, PI19/01796 (FIS Projects), and CD18/00166 [Sara Borrell (for A. Ortega)}, the Spanish Minister of Science, Innovation and Universities [EIN2019-103185], and the European Regional Development Fund.

## DISCLOSURES

No conflicts of interest, financial or otherwise, are declared by the author(s).

## AUTHOR CONTRIBUTIONS

O.M.-A., A.O., J.R., and R.C. conceived and designed research; O.M.-A., A.O., and J.P.-H. performed experiments; O.M.-A., A.O., and R.C. analyzed data; A.O., F.J.C., J.R., and R.C. interpreted results of experiments; A.O. prepared figures; O.M.-A., A.O., and R.C. drafted manuscript; O.M.-A., A.O., J.P.-H., F.J.C., J.R., and R.C. edited and revised manuscript; O.M.-A., A.O., J.P.-H., F.J.C., J.R., and R.C. approved final version of manuscript.

## References

[1] American Diabetes Association (2) Classification and diagnosis of diabetes. Diabetes Care 38, Suppl: S8–S16, 2015. doi:10.2337/dc15-S005. 25537714

[2] Armelloni S, Calvaresi N, Ikehata M, Corbelli A, Mattinzoli D, Giardino LA, Li M, Messa P, Rastaldi MP Proteinuria and glomerular damage in Rab3A knockout mice chronically fed a high-glucose diet. Nephron, Exp Nephrol 120: e69–e80, 2012. doi:10.1159/000336166. 22472623

[3] Bereczki E, Francis PT, Howlett D, Pereira JB, Höglund K, Bogstedt A, Cedazo-Minguez A, Baek JH, Hortobágyi T, Attems J, Ballard C, Aarsland D Synaptic proteins predict cognitive decline in Alzheimer’s disease and Lewy body dementia. Alzheimers Dement 12: 1149–1158, 2016. doi:10.1016/j.jalz.2016.04.005. 27224930

[4] Burns ME, Sasaki T, Takai Y, Augustine GJ Rabphilin-3A: a multifunctional regulator of synaptic vesicle traffic. J Gen Physiol 111: 243–255, 1998. doi:10.1085/jgp.111.2.243. 9450942 PMC2222762

[5] Cummings BS, Schnellmann RG Measurement of cell death in mammalian cells. Curr Protoc Pharmacol Chapter 12: Unit 12.8, 2004. doi:10.1002/0471141755.ph1208s25. 22294120 PMC3874588

[6] Chittiprol S, Chen P, Petrovic-Djergovic D, Eichler T, Ransom RF Marker expression, behaviors, and responses vary in different lines of conditionally immortalized cultured podocytes. Am J Physiol Renal Physiol 301: F660–F671, 2011. doi:10.1152/ajprenal.00234.2011. 21632959 PMC3174553

[7] Chung SH, Takai Y, Holz RW Evidence that the Rab3a-binding protein, rabphilin3a, enhances regulated secretion. Studies in adrenal chromaffin cells. J Biol Chem 270: 16714–16718, 1995. doi:10.1074/jbc.270.28.16714. 7622481

[8] de Sousa Abreu R, Penalva LO, Marcotte EM, Vogel C Global signatures of protein and mRNA expression levels. Mol Biosyst 5: 1512–1526, 2009. doi:10.1039/b908315d. 20023718 PMC4089977

[9] Dorval G, Kuzmuk V, Gribouval O, Welsh GI, Bierzynska A, Schmitt A, Miserey-Lenkei S, Koziell A, Haq S, Benmerah A, Mollet G, Boyer O, Saleem MA, Antignac C TBC1D8B loss-of-function mutations lead to X-linked nephrotic syndrome via defective trafficking pathways. Am J Hum Genet 104: 348–355, 2019. doi:10.1016/j.ajhg.2018.12.016. 30661770 PMC6369567

[10] Eckardt KU, Berns JS, Rocco MV, Kasiske BL Definition and classification of CKD: the debate should be about patient prognosis--a position statement from KDOQI and KDIGO. Am J Kidney Dis 53: 915–920, 2009. doi:10.1053/j.ajkd.2009.04.001. 19406541

[11] Fukuda M Distinct Rab binding specificity of Rim1, Rim2, rabphilin, and Noc2. Identification of a critical determinant of Rab3A/Rab27A recognition by Rim2. J Biol Chem 278: 15373–15380, 2003. doi:10.1074/jbc.M212341200. 12578829

[12] Fukuda M Regulation of secretory vesicle traffic by Rab small GTPases. Cell Mol Life Sci 65: 2801–2813, 2008. doi:10.1007/s00018-008-8351-4. 18726178 PMC11131888

[13] Ghijsen WE, Leenders AG Differential signaling in presynaptic neurotransmitter release. Cell Mol Life Sci 62: 937–954, 2005. doi:10.1007/s00018-004-4525-0. 15761671 PMC11924413

[14] Giardino L, Armelloni S, Corbelli A, Mattinzoli D, Zennaro C, Guerrot D, Tourrel F, Ikehata M, Li M, Berra S, Carraro M, Messa P, Rastaldi MP Podocyte glutamatergic signaling contributes to the function of the glomerular filtration barrier. J Am Soc Nephrol 20: 1929–1940, 2009. doi:10.1681/ASN.2008121286. 19578006 PMC2736779

[15] Grahammer F New structural insights into podocyte biology. Cell Tissue Res 369: 5–10, 2017. doi:10.1007/s00441-017-2590-3. 28283912 PMC5487842

[16] Gu J, Yang M, Qi N, Mei S, Chen J, Song S, Jing Y, Chen M, He L, Sun L, Hu H, Li L, Wüthrich RP, Wu M, Mei C Olmesartan prevents microalbuminuria in *db*/*db* diabetic mice through inhibition of angiotensin II/p38/SIRT1-induced podocyte apoptosis. Kidney Blood Press Res 41: 848–864, 2016. doi:10.1159/000452588. 27871084

[17] Hausser J, Zavolan M Identification and consequences of miRNA-target interactions--beyond repression of gene expression. Nat Rev Genet 15: 599–612, 2014. doi:10.1038/nrg3765. 25022902

[18] Herman-Edelstein M, Thomas MC, Thallas-Bonke V, Saleem M, Cooper ME, Kantharidis P Dedifferentiation of immortalized human podocytes in response to transforming growth factor-β: a model for diabetic podocytopathy. Diabetes 60: 1779–1788, 2011. doi:10.2337/db10-1110. 21521871 PMC3114395

[19] Huang G, Zou B, Lv J, Li T, Huai G, Xiang S, Lu S, Luo H, Zhang Y, Jin Y, Wang Y Notoginsenoside R1 attenuates glucose-induced podocyte injury via the inhibition of apoptosis and the activation of autophagy through the PI3K/Akt/mTOR signaling pathway. Int J Mol Med 39: 559–568, 2017. doi:10.3892/ijmm.2017.2864. 28112381 PMC5360354

[20] Huang L, You YS, Wu W Role of CD2-associated protein in podocyte apoptosis and proteinuria induced by angiotensin II. Ren Fail 36: 1328–1332, 2014. doi:10.3109/0886022X.2014.934177. 24986244

[21] Huang N, Zhang X, Jiang Y, Mei H, Zhang L, Zhang Q, Hu J, Chen B Increased levels of serum pigment epithelium-derived factor aggravate proteinuria via induction of podocyte actin rearrangement. Int Urol Nephrol 51: 359–367, 2019. doi:10.1007/s11255-018-2026-3. 30536192 PMC6394770

[22] Hurcombe JA, Hartley P, Lay AC, Ni L, Bedford JJ, Leader JP, Singh S, Murphy A, Scudamore CL, Marquez E, Barrington AF, Pinto V, Marchetti M, Wong LF, Uney J, Saleem MA, Mathieson PW, Patel S, Walker RJ, Woodgett JR, Quaggin SE, Welsh GI, Coward RJM Podocyte GSK3 is an evolutionarily conserved critical regulator of kidney function. Nat Commun 10: 403, 2019. doi:10.1038/s41467-018-08235-1. 30679422 PMC6345761

[23] Hwang SJ, Yang Q, Meigs JB, Pearce EN, Fox CS A genome-wide association for kidney function and endocrine-related traits in the NHLBI’s Framingham Heart Study. BMC Med Genet 8, Suppl 1: S10, 2007. doi:10.1186/1471-2350-8-S1-S10. 17903292 PMC1995611

[24] Iguchi Y, Eid L, Parent M, Soucy G, Bareil C, Riku Y, Kawai K, Takagi S, Yoshida M, Katsuno M, Sobue G, Julien JP Exosome secretion is a key pathway for clearance of pathological TDP-43. Brain 139: 3187–3201, 2016. doi:10.1093/brain/aww237. 27679482 PMC5840881

[25] Jiang L, Cui H, Ding J Smad3 signalling affects high glucose-induced podocyte injury via regulation of the cytoskeletal protein transgelin. Nephrology (Carlton). In press. doi:10.1111/nep.13701. 32034833 PMC7496067

[26] Kato M, Sasaki T, Ohya T, Nakanishi H, Nishioka H, Imamura M, Takai Y Physical and functional interaction of rabphilin-3A with alpha-actinin. J Biol Chem 271: 31775–31778, 1996. doi:10.1074/jbc.271.50.31775. 8943213

[27] Kiral FR, Kohrs FE, Jin EJ, Hiesinger PR Rab GTPases and membrane trafficking in neurodegeneration. Curr Biol 28: R471–R486, 2018. doi:10.1016/j.cub.2018.02.010. 29689231 PMC5965285

[28] Kuroda TS, Fukuda M, Ariga H, Mikoshiba K The Slp homology domain of synaptotagmin-like proteins 1−4 and Slac2 functions as a novel Rab27A binding domain. J Biol Chem 277: 9212–9218, 2002. doi:10.1074/jbc.M112414200. 11773082

[29] Lang T, Jahn R Core proteins of the secretory machinery. Handb Exp Pharmacol 184: 107–127, 2008. doi:10.1007/978-3-540-74805-2_5. 18064413

[30] Li D, Wang N, Zhang L, Hanyu Z, Xueyuan B, Fu B, Shaoyuan C, Zhang W, Xuefeng S, Li R, Chen X Mesenchymal stem cells protect podocytes from apoptosis induced by high glucose via secretion of epithelial growth factor. Stem Cell Res Ther 4: 103, 2013. doi:10.1186/scrt314. 24004644 PMC3856604

[31] Li S, Liu X, Lei J, Yang J, Tian P, Gao Y Crocin protects podocytes against oxidative stress and inflammation induced by high glucose through inhibition of NF-κB. Cell Physiol Biochem 42: 1481–1492, 2017. doi:10.1159/000479212. 28719912

[32] Li W, Jiang YH, Wang Y, Zhao M, Hou GJ, Hu HZ, Zhou L Protective effects of combination of radix astragali and radix salviae miltiorrhizae on kidney of spontaneously hypertensive rats and renal intrinsic cells. Chin J Integr Med, 2019. 31388973 10.1007/s11655-019-3071-1

[33] Lin RC, Scheller RH Mechanisms of synaptic vesicle exocytosis. Annu Rev Cell Dev Biol 16: 19–49, 2000. doi:10.1146/annurev.cellbio.16.1.19. 11031229

[34] Liu Y, Beyer A, Aebersold R On the dependency of cellular protein levels on mRNA abundance. Cell 165: 535–550, 2016. doi:10.1016/j.cell.2016.03.014. 27104977

[35] Lu XY, Liu BC, Cao YZ, Song C, Su H, Chen G, Klein JD, Zhang HX, Wang LH, Ma HP High glucose reduces expression of podocin in cultured human podocytes by stimulating TRPC6. Am J Physiol Renal Physiol 317: F1605–F1611, 2019. doi:10.1152/ajprenal.00215.2019. 31566428 PMC6960785

[36] Lv Z, Hu M, Ren X, Fan M, Zhen J, Chen L, Lin J, Ding N, Wang Q, Wang R Fyn mediates high glucose-induced actin cytoskeleton reorganization of podocytes via promoting ROCK activation in vitro. J Diabetes Res 2016: 5671803, 2016. doi:10.1155/2016/5671803. 26881253 PMC4736797

[37] Macconi D, Abbate M, Morigi M, Angioletti S, Mister M, Buelli S, Bonomelli M, Mundel P, Endlich K, Remuzzi A, Remuzzi G Permselective dysfunction of podocyte-podocyte contact upon angiotensin II unravels the molecular target for renoprotective intervention. Am J Pathol 168: 1073–1085, 2006. doi:10.2353/ajpath.2006.050701. 16565484 PMC1606571

[38] Maier T, Güell M, Serrano L Correlation of mRNA and protein in complex biological samples. FEBS Lett 583: 3966–3973, 2009. doi:10.1016/j.febslet.2009.10.036. 19850042

[39] Marrachelli VG, Monleon D, Rentero P, Mansego ML, Morales JM, Galan I, Segura R, Martinez F, Martin-Escudero JC, Briongos L, Marin P, Lliso G, Chaves FJ, Redon J Genomic and metabolomic profile associated to microalbuminuria. PLoS One 9: e98227, 2014. doi:10.1371/journal.pone.0098227. 24918908 PMC4053470

[40] National Kidney Foundation K/DOQI clinical practice guidelines for chronic kidney disease: evaluation, classification, and stratification. Am J Kidney Dis 39, Suppl 1: S1–S266, 2002. 11904577

[41] Niranjan T, Bielesz B, Gruenwald A, Ponda MP, Kopp JB, Thomas DB, Susztak K The Notch pathway in podocytes plays a role in the development of glomerular disease. Nat Med 14: 290–298, 2008. doi:10.1038/nm1731. 18311147

[42] Ostrowski M, Carmo NB, Krumeich S, Fanget I, Raposo G, Savina A, Moita CF, Schauer K, Hume AN, Freitas RP, Goud B, Benaroch P, Hacohen N, Fukuda M, Desnos C, Seabra MC, Darchen F, Amigorena S, Moita LF, Thery C Rab27a and Rab27b control different steps of the exosome secretion pathway. Nat Cell Biol 12: 19−13, 2010. doi:10.1038/ncb2000. 19966785

[43] Perez-Hernandez J, Olivares MD, Forner MJ, Chaves FJ, Cortes R, Redon J Urinary dedifferentiated podocytes as a non-invasive biomarker of lupus nephritis. Nephrol Dial Transplant 31: 780–789, 2016. doi:10.1093/ndt/gfw002. 26932688

[44] Perez-Hernandez J, Olivares MD, Solaz E, Martinez F, Martínez-Hervas S, Pichler G, Chaves FJ, Redon J, Cortes R Urinary podocyte-associated molecules and albuminuria in hypertension. J Hypertens 36: 1712–1718, 2018. doi:10.1097/HJH.0000000000001747. 29677049

[45] Perico L, Conti S, Benigni A, Remuzzi G Podocyte-actin dynamics in health and disease. Nat Rev Nephrol 12: 692–710, 2016. doi:10.1038/nrneph.2016.127. 27573725

[46] Potter AS, Drake K, Brunskill EW, Potter SS A bigenic mouse model of FSGS reveals perturbed pathways in podocytes, mesangial cells and endothelial cells. PLoS One 14: e0216261, 2019. doi:10.1371/journal.pone.0216261. 31461442 PMC6713350

[47] Pylypenko O, Hammich H, Yu IM, Houdusse A Rab GTPases and their interacting protein partners: Structural insights into Rab functional diversity. Small GTPases 9: 22–48, 2018. doi:10.1080/21541248.2017.1336191. 28632484 PMC5902227

[48] Quevedo MF, Bustos MA, Masone D, Roggero CM, Bustos DM, Tomes CN Grab recruitment by Rab27A-Rabphilin3a triggers Rab3A activation in human sperm exocytosis. Biochim Biophys Acta Mol Cell Res 1866: 612–622, 2019. doi:10.1016/j.bbamcr.2018.12.005. 30599141

[49] Rastaldi MP, Armelloni S, Berra S, Calvaresi N, Corbelli A, Giardino LA, Li M, Wang GQ, Fornasieri A, Villa A, Heikkila E, Soliymani R, Boucherot A, Cohen CD, Kretzler M, Nitsche A, Ripamonti M, Malgaroli A, Pesaresi M, Forloni GL, Schlöndorff D, Holthofer H, D’Amico G, Rastaldi MP, Armelloni S, Berra S, Calvaresi N, Corbelli A, Giardino LA, Li M, Wang GQ, Fornasieri A, Villa A, Heikkila E, Soliymani R, Boucherot A, Cohen CD, Kretzler M, Nitsche A, Ripamonti M, Malgaroli A, Pesaresi M, Forloni GL, Schlöndorff D, Holthofer H, D’Amico G Glomerular podocytes contain neuron-like functional synaptic vesicles. FASEB J 20: 976–978, 2006. doi:10.1096/fj.05-4962fje. 16585060

[50] Rastaldi MP, Armelloni S, Berra S, Li M, Pesaresi M, Poczewski H, Langer B, Kerjaschki D, Henger A, Blattner SM, Kretzler M, Wanke R, D’Amico G Glomerular podocytes possess the synaptic vesicle molecule Rab3A and its specific effector rabphilin-3a. Am J Pathol 163: 889–899, 2003. doi:10.1016/S0002-9440(10)63449-9. 12937130 PMC1868247

[51] Saleem MA, O’Hare MJ, Reiser J, Coward RJ, Inward CD, Farren T, Xing CY, Ni L, Mathieson PW, Mundel P A conditionally immortalized human podocyte cell line demonstrating nephrin and podocin expression. J Am Soc Nephrol 13: 630–638, 2002. 11856766 10.1681/ASN.V133630

[52] Sanchez-Niño MD, Sanz AB, Sanchez-Lopez E, Ruiz-Ortega M, Benito-Martin A, Saleem MA, Mathieson PW, Mezzano S, Egido J, Ortiz A HSP27/HSPB1 as an adaptive podocyte antiapoptotic protein activated by high glucose and angiotensin II. Lab Invest 92: 32–45, 2012. doi:10.1038/labinvest.2011.138. 21931298

[53] Scott RP, Quaggin SE Review series: The cell biology of renal filtration. J Cell Biol 209: 199–210, 2015. doi:10.1083/jcb.201410017. 25918223 PMC4411276

[54] Schlüter OM, Schmitz F, Jahn R, Rosenmund C, Südhof TC A complete genetic analysis of neuronal Rab3 function. J Neurosci 24: 6629–6637, 2004. doi:10.1523/JNEUROSCI.1610-04.2004. 15269275 PMC6729882

[55] Schwartz SL, Cao C, Pylypenko O, Rak A, Wandinger-Ness A Rab GTPases at a glance. J Cell Sci 120: 3905–3910, 2007. doi:10.1242/jcs.015909. 17989088

[56] Seong SB, Ha DS, Min SY, Ha TS Autophagy precedes apoptosis in angiotensin II-induced podocyte injury. Cell Physiol Biochem 53: 747–759, 2019. doi:10.33594/000000170. 31622062

[57] Shankland SJ The podocyte’s response to injury: role in proteinuria and glomerulosclerosis. Kidney Int 69: 2131–2147, 2006. doi:10.1038/sj.ki.5000410. 16688120

[58] Shaw JE, Sicree RA, Zimmet PZ Global estimates of the prevalence of diabetes for 2010 and 2030. Diabetes Res Clin Pract 87: 4–14, 2010. doi:10.1016/j.diabres.2009.10.007. 19896746

[59] Simons M, Saffrich R, Reiser J, Mundel P Directed membrane transport is involved in process formation in cultured podocytes. J Am Soc Nephrol 10: 1633–1639, 1999. 10446930 10.1681/ASN.V1081633

[60] Song L, Tang S, Han X, Jiang Z, Dong L, Liu C, Liang X, Dong J, Qiu C, Wang Y, Du Y KIBRA controls exosome secretion via inhibiting the proteasomal degradation of Rab27a. Nat Commun 10: 1639, 2019. doi:10.1038/s41467-019-09720-x. 30967557 PMC6456494

[61] Stettler O, Moya KL, Zahraoui A, Tavitian B Developmental changes in the localization of the synaptic vesicle protein rab3A in rat brain. Neuroscience 62: 587–600, 1994. doi:10.1016/0306-4522(94)90391-3. 7830899

[62] Succar L, Boadle RA, Harris DC, Rangan GK Formation of tight junctions between neighboring podocytes is an early ultrastructural feature in experimental crescentic glomerulonephritis. Int J Nephrol Renovasc Dis 9: 297–312, 2016. doi:10.2147/IJNRD.S113071. 27920570 PMC5126005

[63] Suh JH, Miner JH The glomerular basement membrane as a barrier to albumin. Nat Rev Nephrol 9: 470–477, 2013. doi:10.1038/nrneph.2013.109. 23774818 PMC3839671

[64] Tian J, Zhang L, Zhou Y, Xiao J, Li S, Chen Y, Qiao Z, Niu J, Gu Y Angiotensin-(1−7) attenuates damage to podocytes induced by preeclamptic serum through MAPK pathways. Int J Mol Med 34: 1057–1064, 2014. doi:10.3892/ijmm.2014.1870. 25092178

[65] Tsuboi T, Fukuda M Rab3A and Rab27A cooperatively regulate the docking step of dense-core vesicle exocytosis in PC12 cells. J Cell Sci 119: 2196–2203, 2006. doi:10.1242/jcs.02962. 16684812

[66] Vogel C, Marcotte EM Insights into the regulation of protein abundance from proteomic and transcriptomic analyses. Nat Rev Genet 13: 227–232, 2012. doi:10.1038/nrg3185. 22411467 PMC3654667

[67] Williams B, Mancia G, Spiering W, Agabiti Rosei E, Azizi M, Burnier M, Clement DL, Coca A, de Simone G, Dominiczak A, Kahan T, Mahfoud F, Redon J, Ruilope L, Zanchetti A, Kerins M, Kjeldsen SE, Kreutz R, Laurent S, Lip GYH, McManus R, Narkiewicz K, Ruschitzka F, Schmieder RE, Shlyakhto E, Tsioufis C, Aboyans V, Desormais I; ESC Scientific Document Group 2018 ESC/ESH guidelines for the management of arterial hypertension. Eur Heart J 39: 3021–3104, 2018. doi:10.1093/eurheartj/ehy339. 30165516

[68] Wolf G, Chen S, Ziyadeh FN From the periphery of the glomerular capillary wall toward the center of disease: podocyte injury comes of age in diabetic nephropathy. Diabetes 54: 1626–1634, 2005. doi:10.2337/diabetes.54.6.1626. 15919782

[69] World Medical Association World Medical Association Declaration of Helsinki: ethical principles for medical research involving human subjects. JAMA 310: 2191–2194, 2013. doi:10.1001/jama.2013.281053. 24141714

[70] Yasuda T, Saegusa C, Kamakura S, Sumimoto H, Fukuda M Rab27 effector Slp2-a transports the apical signaling molecule podocalyxin to the apical surface of MDCK II cells and regulates claudin-2 expression. Mol Biol Cell 23: 3229–3239, 2012. doi:10.1091/mbc.e12-02-0104. 22767581 PMC3418316

[71] Zhen Y, Stenmark H Cellular functions of Rab GTPases at a glance. J Cell Sci 128: 3171–3176, 2015. doi:10.1242/jcs.166074. 26272922

